# Hybrid Hydrogen Sensor Based on Pd/WO_3_ Showing Simultaneous Chemiresistive and Gasochromic Response

**DOI:** 10.3390/nano13182563

**Published:** 2023-09-15

**Authors:** Sanghoon Kim, Bohee Maeng, Yijun Yang, Kwanwoo Kim, Daewoong Jung

**Affiliations:** Advanced Mechatronics R&D Group, Korea Institute of Industrial Technology (KITECH), Daegu 42994, Republic of Korea; kimsanghoon@kitech.re.kr (S.K.); zriko323@kitech.re.kr (B.M.); yjyang@kitech.re.kr (Y.Y.); les6085@kitech.re.kr (K.K.)

**Keywords:** gasochromic hydrogen sensor, hybrid hydrogen sensor, hydrogen sensor, gasochromic hybrid gas sensor, gasochromic hybrid hydrogen sensor

## Abstract

The gasochromism of WO_3_, wherein the color of the material changes according to the reaction of gas, can immediately allow for the determination of the presence of hydrogen by the naked eye. We have also developed a hybrid hydrogen sensor for WO_3_, a metal oxide, that can simultaneously utilize its gasochromic response and resistance to hydrogen. Because the proposed sensor has a transparent electrode on a glass substrate, it is a structure that can not only reveal the change in resistance but also more clearly illustrate the gasochromic response. A hybrid sensing demonstration in a hydrogen leak environment was successfully performed to verify a sensor that was capable of utilizing the resistive and gasochromic response of WO_3_.

## 1. Introduction

Unlike conventional fossil fuel energy sources, hydrogen has been considered to be a promising alternative energy source in various applications such as automobiles, internal combustion engines, and battery cells because it is environmentally friendly and can be obtained from water [1]. However, because hydrogen has a lower explosive limit at a low concentration of 4%, its flammability and explosiveness are quite high [2]. It is colorless, odorless, and diffuses very quickly; these factors make it very difficult for humans to detect hydrogen leaks, which can potentially lead to serious accidents [3]. Therefore, hydrogen sensors are essential in some areas of industries as it is very important to quickly detect leaks of hydrogen at low concentrations to prevent explosion hazards [4].

To use hydrogen, the use of a hydrogen detection sensor is essential, and appropriate standards must be determined with the aim of operating safely and efficiently in the use environment, including in the production method and processing of hydrogen; the storage method; the transportation means; and the use method [5]. The National Renewable Energy Laboratory (NREL) of the US Department of Energy (DOE) provides safety requirements and background information on hydrogen to various stakeholders that handle hydrogen, along with guidelines on how to safely handle hydrogen [6].

Some of the most widely used hydrogen sensor types include semiconductor-type [7], catalytic combustion-type [8], field effect transistor (FET)-type [9], electrolyte-type (electrochemical type) [10], optical-type [11], piezo-type [12], thermo-type [13], and gasistor-type [14] sensors. The selectivity, sensitivity, response time, reproducibility, long-term stability, lifetime, and reversibility of these sensors can be determined by the concentration, operating temperature, pressure, and interference effects of impurities. For this reason, cases of applying catalytic metals [15,16] or hetero-structured [17,18] gas sensor have been reported to improve the sensitivity or reaction rate of the sensor.

An optical sensor that uses a gasochromic material, whose light transmittance changes according to hydrogen adsorption, does not require a direct flow of electricity into the sensor; therefore, there is no risk of hydrogen explosion due to electric discharge, which means that this method can solve the drawbacks of existing sensors [19]. With the gasochromic method, hydrogen leaks can be detected remotely through light transmitted from the sensing material to the optical spectrometer, or immediately to the human eye without optical spectrometer due to the color change in visible light wavelengths. Remote hydrogen detection is possible, and immediate detection is possible with the human eye without a signal converter in the sensor circuit due to the color change in the visible wavelength. The advantage of the gaschromic sensor is that it fundamentally blocks the possibility of explosion due to discharge because only light is sent without an electrical signal flowing to the part that is exposed to hydrogen [20]. In addition, because it is designed to transmit and receive probe beams using an optical fiber, remote hydrogen detection is possible through the separation of the sensor and the signal processing unit [20]. To increase sensitivity and improve durability, many researchers have been making efforts to develop electrochromic oxides such as tungsten trioxide (WO_3_), whose light transmittance is greatly changed by hydrogen, as gasochromic sensors [21,22].

The chemiresistive-type sensor which is the most widely used gas sensor type is very complex and requires many design elements [7]. On the other hand, the gasochromic-type hydrogen sensor detects the change in color with the naked eye so that the user can qualitatively detect hydrogen leakage [23]. To take only the advantages of each type of sensors, there were some cases of simultaneous observations of color change and electrical signals by using different sensing materials respectively for gasochromic and chemiresistive response in the same sensor platform [24,25].

WO_3_ is a representative gasochromic material and is well-known to exhibit coloration by adsorbing hydrogen [20,21,22]. It shows a reversible colored state and bleached state depending on the adsorbed hydrogen [20]. Because gasochromic performance deteriorates due to amorphous crystallization, several researchers have reported research on crystalline WO_3_ in order to improve the discoloration performance [23]. In addition, a WO_3_ thin film in the form of a porous structure has a large surface area, has low resistance to charging and dissociated hydrogen diffusion, and shows high charge mobility [23,24].

In this paper, we propose a hybrid-type hydrogen sensor that can simultaneously utilize the gasochromic reaction and chemiresistive properties of WO_3_. Palladium (Pd), a catalytic metal that selectively adsorbs hydrogen and effectively dissociates it to the substrate [26], is applied, and the electrode for observing the resistance change of the WO_3_ layer deposited on the glass substrate is formed of indium tin oxide (ITO), a highly permeable transparent electrode. Due to this designed structure, changes in color can be effectively observed. Because the chemical behavior of a single substance can be observed in two ways, the structure is simple compared with methods in which each type of sensor is separately used [24,25].

## 2. Experimental Details

### 2.1. Fabrication of Proposed Hydrogen Sensor

**Figure 1:**
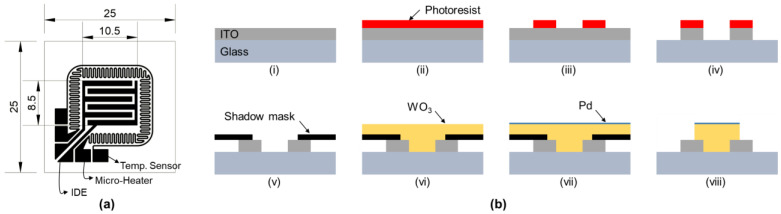
(**a**) Design of the IDEs, microheater, and temperature sensor pattern (unit: mm). (**b**) Fabrication process of the proposed hybrid-type hydrogen sensor.

A highly permeable substrate was used so that the gasochromic reaction of WO_3_ could be visually confirmed by humans. The fabrication process of the sensor can be seen in Figure 1b; ITO was deposited at 1500 Ȧ on non-alkali glass with a thickness of 700 μm. ITO has a resistance of less than 10 Ω/sq and a transmittance of more than 85% (λ = 550 nm). The inter-digitated electrodes (IDEs) to be used as electrodes in the sensing area, the microheater pattern to control the operating temperature of the sensing material, and the temperature sensor were patterned using the dry etching technique. Then, WO_3_ and Pd were deposited at 400 nm and 5 nm, respectively, in the region excluding the electrode pads using a shadow mask. WO_3_ and Pd were deposited using DC magnetron sputtering and E-beam deposition, respectively. To ensure moderate deposition of WO_3_ by the sputtering, a power of 150 W was carried out in a 99.99% Ar atmosphere at a flow rate of 20 sccm, and the deposition rate was set to about 0.8 Ȧ/s. Additionally, Pd deposition using an E-beam had a power ranging from 7 to 15 mW and a deposition rate ranging from 2 to 4 Ȧ/s. Both WO_3_ and Pd were deposited at 25 °C, and after the deposition process was completed, a performance evaluation was conducted. To ensure the stable crystallization of the oxide film, annealing was performed at 300 °C for 3 h each under Ar and air conditions at 1 atm. Figure 1a is the design drawing of the IDEs, micro-heater, and temperature sensor that was patterned on the ITO layer. The cell size of the sensor is 25 mm^2^, and the sensing area is 8.5 mm × 10.5 mm. Figure 1b (i–viii) shows the step-by-step process of the fabrication process described above.

### 2.2. Characterization

**Figure 2:**
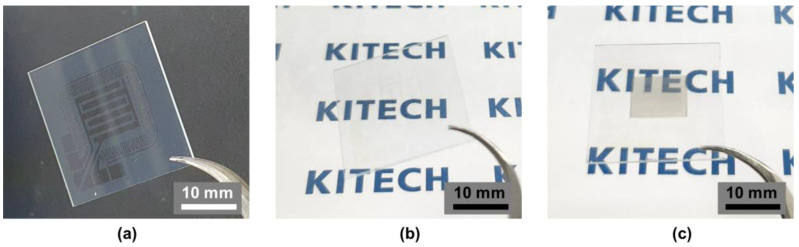
(**a**,**b**) ITO-patterned glass cell and (**c**) fabricated hydrogen sensor after WO_3_ and Pd deposition.

**Figure 3:**
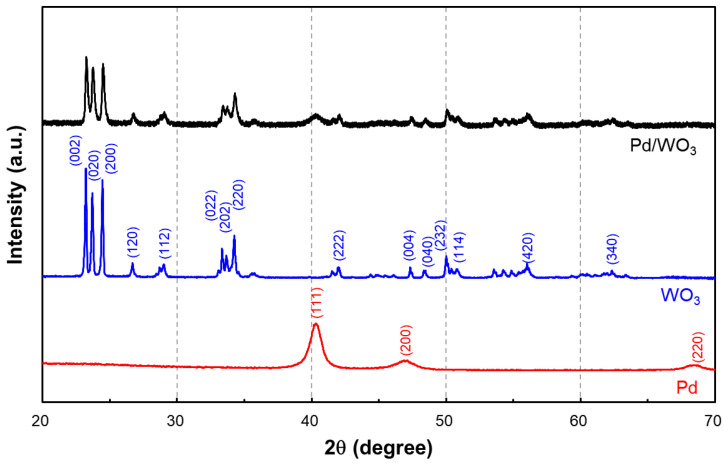
X-ray diffraction (XRD) patterns of WO_3_, Pd, and the Pd/WO_3_ sensing layer.

Figure 2a,b shows the ITO-patterned glass. As shown in Figure 2b, it shows a very high transmittance due to the very high transmittance of the patterned ITO layer, and Figure 2c is a picture of the hydrogen sensor as completed by depositing WO_3_ and Pd. The sensing area where Pd is deposited on WO_3_ can be seen as a slight yellow to off-white color. The crystal structure and purity of the WO_3_ and Pd that was deposited on the sensing layer were investigated using X-ray diffraction (XRD), as shown in Figure 3. Then, field emission scanning electron microscopy (FE-SEM) images and energy-dispersive spectrometer (EDS) analyses of the cross-section of the sensing layer composed of Pd-coated WO_3_ were conducted. In addition, the surface roughness was analyzed using an atomic force microscope (AFM) to observe the structure of the Pd/WO_3_ that was deposited on the surface of the ITO electrode on the glass substrate.

Figure 3 shows the X-ray diffraction patterns for WO_3_, Pd, and Pd/WO_3_. As shown in the figure, the peaks at 2θ = 22.9, 23.4, 24.1, 26.4, 28.1,32.8, 33.1, 33.9, 41.6, 49.82, 50.43, 55.6, and 62.0° corresponded to the (002), (020), (200), (120), (112), (022), (202), (220), (222), (004), (040), (232), (114), (420), and (340) crystal faces of WO_3_ (blue line), respectively, which is consistent with the earlier reported literature and JCPDS #83-0950. Furthermore, it appears that two of the strongest peaks at 2θ = 40.1 and 46.6° for Pd were the characteristic (111) and (200) peaks of Pd (red line), which is consistent with JCPDS #05-0681. In Pd/WO_3_, the peaks of WO_3_ and Pd were overlapped (black line). Clear diffraction peaks were assigned to the orthorhombic WO_3_ structure. Additionally, peaks of Pd particles were also observed in the diffraction spectrum of Pd/WO_3_, indicating that Pd was successfully loaded onto the WO_3_ surface. The appearance of Pd diffraction peaks indicates that the experimentally deposited palladium is supported on the WO_3_ surface.

**Figure 4:**
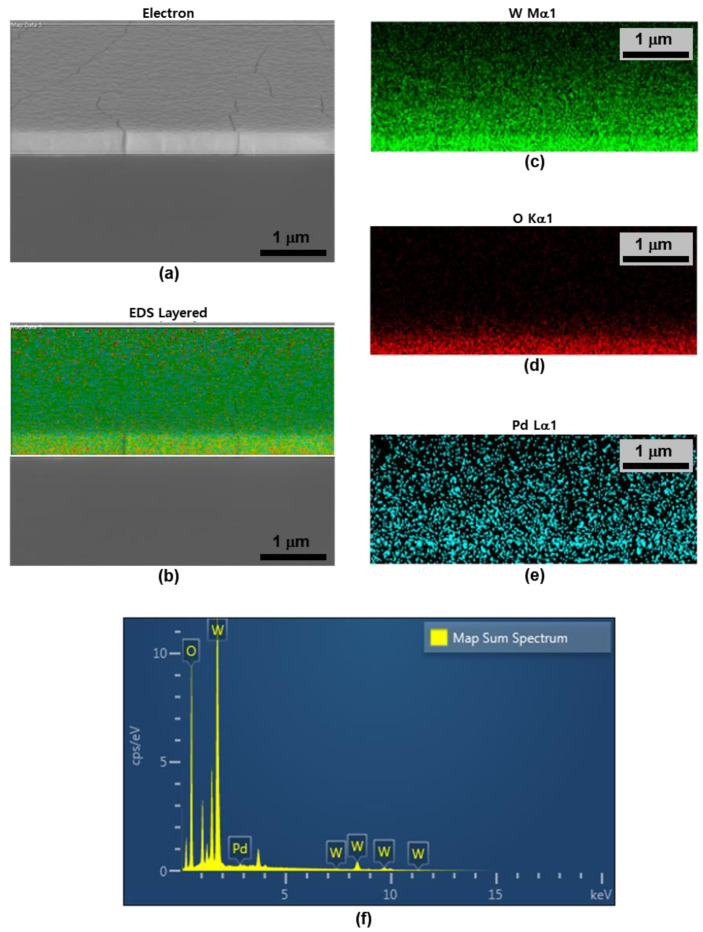
Results of analyzing the surface shape and constituent elements of the fabricated Pd/WO_3_ through (**a**) FE-SEM, (**b**) element EDS layered on an electron image, and EDS element mapping for (**c**) W, (**d**) O, and (**e**) Pd. (**f**) EDS spectra of the Pd/WO_3_ sensing layer.

Figure 4 shows the results of analyzing the surface shape and constituent elements of the fabricated Pd/WO_3_ using FE-SEM and EDS element mapping. The figure shows (a) an electron image of the tilted Pd/WO_3_ surface; (b) the element EDS layered on an electron image; and EDS mapping for (c) W, (d) O, and (e) Pd, respectively. As shown in Table 1 and Figure 4f, the composition consisted of W, O, and Pd at amounts of 50.54, 48.04, and 1.43 wt%, respectively. When comparing Figure 4d with the electron image, the atomic intensity of Pd is revealed based on the shape of the structure of the WO_3_ surface. EDS mapping was performed on the cross-section at a tilted angle to confirm that a thin Pd coat had been applied to the surface of WO_3_.

**Figure 5:**
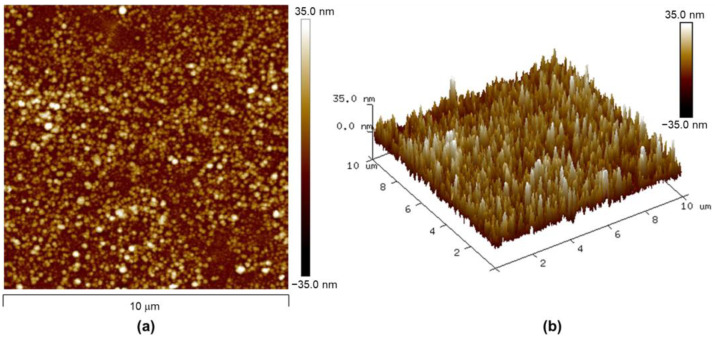
(**a**,**b**) AFM image of the Pd/WO_3_ surface.

The surface topography of the processed Pd/WO_3_ surface was investigated by using an AFM in noncontact mode. Figure 5a,b shows the 2D and 3D AFM images (investigation area size = 10 μm^2^) of the Pd/WO_3_ surface. The roughness average (R_a_) and root mean square roughness (R_q_) were 6.97 and 8.74 nm, respectively. Amorphous WO_3_ thin films are an inhibitor of gasochromic and chemiresistive responses [27]. For the improved gasochromic and chemiresistive response of WO_3_, the high specific surface area due to the crystalline and porous structures is very advantageous. High crystallinity was confirmed in the XRD analysis results in Figure 3, and high specific surface area due to the micropore structure was confirmed in Figure 5b. Due to the roughness of the surface, a very porous extended specific surface area was secured, and thus, an improvement in response speed can be expected [28].

ITO, the transparent electrode that we propose applying to the sensor, has an *R_pv_* (roughness; peak-to-valley of the line) of 150 Ȧ and is coated with the WO_3_ thin film and Pd nanoparticles. Due to porous WO_3_ that was sputtered at a low deposition rate at room temperature and nano-catalyst metal coating having higher crystallinity, the electrode showed a rapid gasochromic response to hydrogen. Additionally, it showed a rate of change in the resistance to hydrogen at a level where a signal could be detected even at room temperature. Moreover, the electrode has a structure that can be adjusted to a specific operating temperature according to the user’s intention. It is expected that a higher relative resistance change can be achieved by applying a nanostructure that further improves the surface area.

### 2.3. Experimental Setup

**Figure 6:**
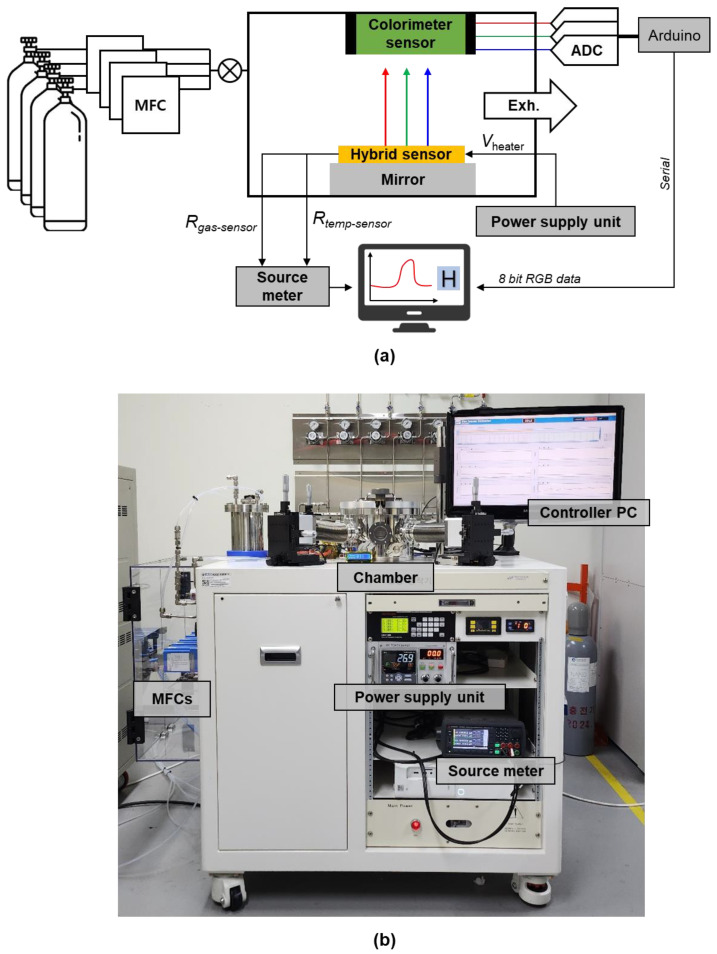
(**a**) Schematic illustration of the experimental setup for measuring the gasochromic-resistive hybrid hydrogen sensor and (**b**) photograph of the experimental setup.

A photograph of the measurement setup is shown in Figure 6. The fabricated device was placed in a sealed gas chamber with a probe station and colorimeter sensor. Hydrogen was diluted with synthetic air, and synthetic air was used as the carrier gas. The gas flow and concentration were controlled by a mass flow controller (MFC; Mykrolis Corp. (Austin, TX, USA), Tylan FC-280SAV). The temperature was precisely controlled by adjusting the applied voltage of the heater to the power supply unit (PSU; Yokogawa Electric Corp. (Musashino, Tokyo, Japan), UT35A) by feeding back the value of the integrated temperature sensor of the device. The resistance change of the sensor was measured using a source meter (Keysight Technologies Inc. (Santa Rosa, CA, USA), B2902B) with a 1 V bias voltage. A colorimeter sensor (AMS Corp. (Knoxville, TN, USA), TCS-34725) was used to measure the gasochromic change of the sensor. We recorded the RGB value measured by the colorimeter sensor and the resistance value measured by the source meter in the computer. The resistance value and color of the sensor can be monitored in real time through the computer’s data-logging software (sweep-me.net, SweepMe! 1.5.5 [29]).

### 2.4. Gasochromic Reactivity Analysis

The proposed sensor was developed for the purpose of remotely monitoring the color change in the visible light wavelength by the user or by detecting the sensor signal. The CIELAB color space was used to confirm the color difference of the sensor before and after the gas reaction. A delta E (ΔE) is a value that numerically represents the difference between two colors distinguished by human visual perception [30]. The value of ΔE at least 3.5 or more, which indicates that color differences can be distinguished by inexperienced observers [25], and it was calculated by the CIELAB equation. Equation (1):

(1) $$\Delta E=\sqrt{[{\left(L_{1}-L_{2}\right)}^{2}+{{\left(a_{1}-a_{2}\right)}^{2}+(b_{1}-b_{2})}^{2}}]$$

where *L* represents the lightness of the color, and a and b are the hue positions between redness and greenness and yellowness and blueness, respectively. As explained in the experimental setup, the fabricated sensor recorded RGB values on the surface of the sensor through a colorimeter sensor. The recorded RGB raw values were converted into an L*a*b* coordinate system using MATLAB (MathWorks, R2023a version) software.

## 3. Results

### 3.1. Working Principles of the Hybrid Hydrogen Sensor

We proposed a hybrid sensor that shows resistance and color change according to the amount of surface charge of WO_3_, a gasochromic MOS material. The hybrid gas sensor was fabricated by depositing a very thin layer of Pd, a catalytic metal that enhances the selective molecular adsorption and dissociation of hydrogen, on WO_3_, an MOS. Pd, a catalytic metal that shows selective adsorption and dissociation of hydrogen (spill-over effect [26]), was very thinly coated on the surface of WO_3_. As a result, the amount of change in the resistance and coloring was improved and a faster response was induced.

WO_3_ is a representative n-type MOS. At high temperature, oxygen is adsorbed on the surface and electrons are reduced from the surface of WO_3_, resulting in a negative charge. In this process, an electron depletion layer is formed on the surface of WO_3_, and the range of redox behavior can be expanded depending on the thickness of the electron depletion layer. Hydrogen combines with the adsorbed oxygen on the WO_3_ surface and is removed, and the electron depletion layer within the WO_3_ particle becomes thinner, so the resistance decreases [21].

WO_3_ is also known as a gasochromic material, and gasochromism is a phenomenon in which the color of a substance changes according to the number of positive ions or electrons. When the surface of WO_3_ is exposed to hydrogen, the light absorption of the oxide increases. The hydrogen atoms dissociated by Pd dissolve in the WO_3_ film and react with oxygen on the surface of the oxide film, leading to water and oxygen vacancies. When oxygen vacancies spread to the inside of WO_3_, tungsten bronze (H*x*WO_3_) is formed due to oxygen deficiency, coloring the film [22]. Through this process, the color of the WO_3_ film becomes bluish.

### 3.2. Exploration of Structure and Operating Conditions

**Figure 7:**
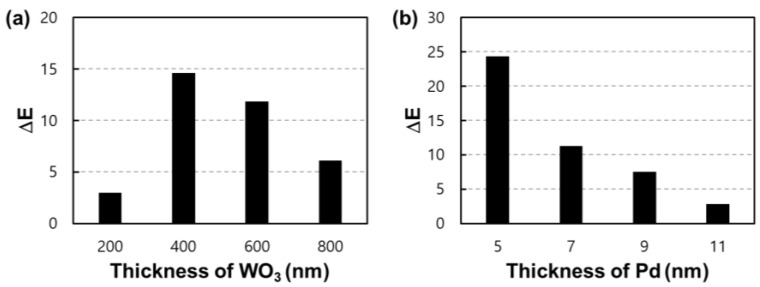
ΔE values for hydrogen at a concentration of 2.5%. (**a**) Color change according to the thickness of the WO_3_ layer coated with 5 nm of Pd and (**b**) ΔE values of the sensor according to the thickness of Pd deposited on 400 nm of WO_3_.

WO_3_ was deposited on the IDE pattern using a DC magnetron sputter. Hydrogen reactivity was compared by depositing 5 nm of Pd on each fabricated sensor by adjusting the thickness of WO_3_. Figure 7 is the result of the experiment conducted at room temperature (25 °C). As shown in Figure 7a, the sensor applied with 400 nm of WO_3_ showed a high change of ΔE = 14.6 for a 2.5% hydrogen concentration. However, it was confirmed that the color change gradually decreased when the thickness became thicker or when the thickness was less than 400 nm. Figure 7b shows the ΔE comparison results observed by adjusting the thickness of Pd that was coated on the sample deposited with 400 nm of WO_3_, which showed the highest reactivity. In the sample that was coated with Pd as thinly as 5 nm, a more improved amount of change was observed with a value of ΔE = 24.32, but as the thickness of Pd increased, the ΔE gradually decreased. For this reason, we considered that Pd forming a film on the WO_3_ layer does not effectively diffuse hydrogen into the WO_3_ layer and adsorbs hydrogen on its own [26]. As a result of this, subsequent experiments were conducted with a sample in which a very thin coating of Pd of 5 nm or less was applied to 400 nm of WO_3_.

### 3.3. Evaluation of the Reactivity of the Fabricated Sensor to Hydrogen

**Figure 8:**
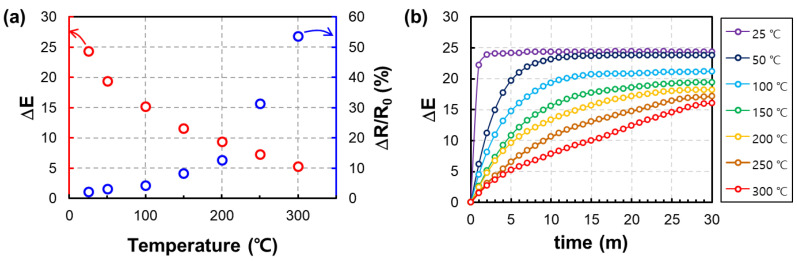
A graph of the reactivity of the fabricated sensor to 2.5% hydrogen at different operating temperatures. (**a**) Color change due to a gasochromic reaction and relative resistance change due to a chemiresistive reaction are indicated by red and blue markers, respectively. (**b**) Gasochromic transient graph at various temperatures over time.

In the previous results, the resistivity and gasochromic response according to the operating temperature of the sensor were confirmed using the optimized structure of the sensor. As shown in Figure 8a, like the general MOS sensor, the relative resistance change has a proportional effect on the rising operating temperature. However, as the temperature increases, the gasochromic response of WO_3_ appears to be decreased. In the reports of several related studies [31,32], it has been reported that the gasochromic reaction has little effect in the temperature range of about 90 °C or more. The result in Figure 8a depicts that the color difference recorded at the time of reacting for 300 s with a 2.5% hydrogen concentration to the gas sensor sufficiently preheated to each temperature. In the case of a longer reaction time (>30 min), as shown in Figure 8b, all are saturated with similar color change values, so the gasochromic response time of the Pd/WO_3_ hybrid sensor can be inferred from this result.

**Figure 9:**
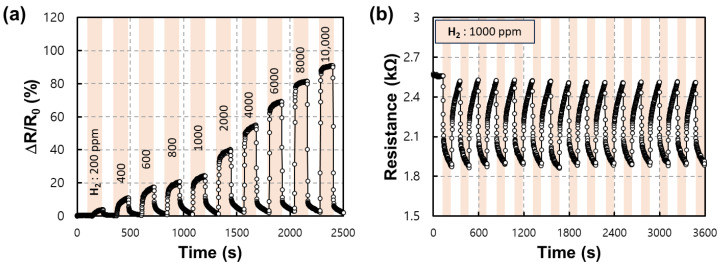
(**a**) Transient response graph for various hydrogen concentrations measured at an operating temperature of 300 °C and (**b**) repeatability measurement graph for the same concentration.

Figure 9a,b shows the response of the fabricated sensor to the adjusted hydrogen concentration. All experiments were conducted at an operating temperature of 300 °C, and hydrogen gas diluted with synthetic air was used. In Figure 9a, a linear response was shown for the elevated hydrogen concentration, and a relative resistance change of about 90% was shown for a maximum H_2_ concentration of 1%. As described above, WO_3_ shows the characteristics of an n-type MOS in which the resistance decreases when reduced by hydrogen. Thus, the relative resistance change was calculated using the formula below for the measured resistance value, Equation (2):

(2) $$Relative\;resistance\;change=\frac{\left|\Delta R\right|}{R_{0}}\times 100\;(\%)$$

where Δ*R*, *R_s_*, and *R*_0_ represents the $R_{s}-R_{0}$, resistance of the gas sensors before (*R*_0_) and after (*R_s_*) exposure to H_2_ gas, respectively. Additionally, in Figure 9b, the stable repeatability for the resistive behavior of the sensor can be confirmed for repeated 1000 ppm of hydrogen gas. Hydrogen and synthetic air were cycled about 15 times to confirm repeatability of the device.

**Figure 10:**
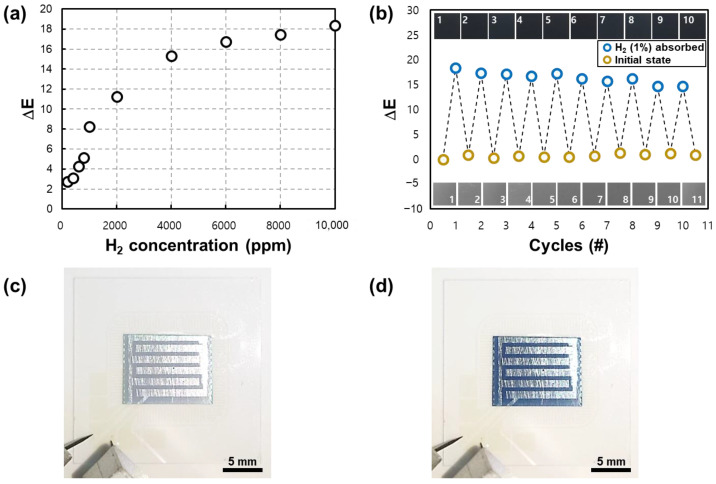
(**a**) Graph of the ΔE values of the sensor according to various hydrogen concentrations and (**b**) ΔE values of repeated results of coloring and bleaching conditions for hydrogen. (**c**) Photograph of the sensor before exposure (bleached state) and (**d**) after exposure to a 1% concentration of hydrogen (colored state).

Figure 10 is the result of the experiment that was conducted to quantify the gasochromism of WO_3_. The experiment was conducted at room temperature (25 °C), and the ΔE in the saturated state was calculated by maintaining the controlled hydrogen concentration. In Figure 10a, the amount of color change that was proportional to the increasing hydrogen concentration was confirmed, and ΔE = 18.4 was confirmed for 1% hydrogen concentration. Moreover, Figure 10b shows the sensor’s color change repetition response. Inside Figure 10b, pictures of the initial state, colored, and bleached surface of the actual sensor are inserted. In repetitive coloring and bleaching cycles, a slight degradation of the amount of change and saturation level was confirmed; however, stable gasochromic behavior was shown with an error range of less than 5% in more than 10 cycles. Figure 10c,d are photographs of the initial state of each fabricated sensor and the colored state through exposure to 1% hydrogen concentration, respectively. The initial state is slightly yellow or bright grayish-white to the naked eye, and when exposed to hydrogen, it becomes very dark blue or navy.

**Figure 11:**
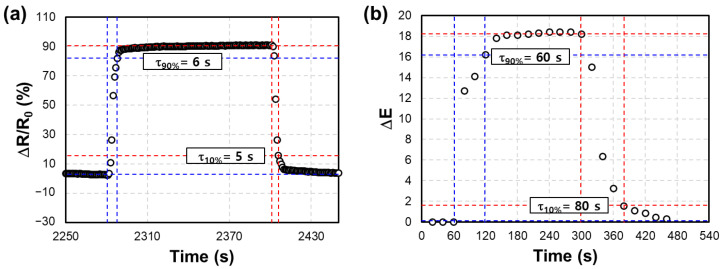
Response (τ_90%_) and recovery (τ_10%_) analysis of (**a**) the resistance and (**b**) gasochromic response for H_2_ at 1% concentration.

Figure 11 is the result of analyzing the resistance- and gasochromic-type response speed of the proposed hybrid hydrogen sensor. The reaction time (τ_90%_) for the initial resistance value (*R*_0_) to reach the 90% point of the saturation resistance value (*R_s_*) after exposure to hydrogen and the recovery time (τ_10%_) for reaching the 10% point of the initial resistance value (*R*_0_) were measured. Figure 11a is the response to 1% hydrogen concentration at 300 °C, and the response time and recovery time were confirmed to be 6 and 5 s, respectively. Figure 11b is the gasochromic reaction to 1% hydrogen concentration at room temperature (25 °C), and the reaction time and recovery time were confirmed to be 60 and 80 s, respectively. Even after the time when the resistance value of the sensor is saturated, the gasochromism of WO_3_ can continue, and the resistance change shows a relatively faster response speed.

**Figure 12:**
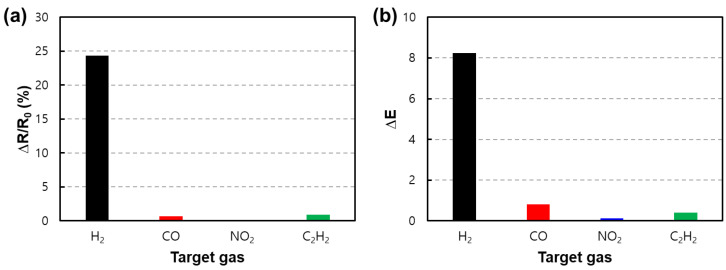
Comparison of reactivity to various gases for the comparison of the hydrogen selectivity of fabricated sensors. Comparison of (**a**) the relative resistance change and (**b**) ΔE for gases at various concentrations of 1000 ppm.

In addition, the fabricated sensor was optimized for the material, catalyst, and operating temperature of the sensor to detect hydrogen. Based on this design, to compare the selective reactivity to hydrogen, a reactivity experiment was conducted by controlling the same operating temperature (300 °C) and concentration of various gases (1000 ppm), as shown in Figure 12. The relative resistance change and ΔE of the fabricated sensor can be confirmed in Figure 12a,b, respectively.

**Figure 13:**
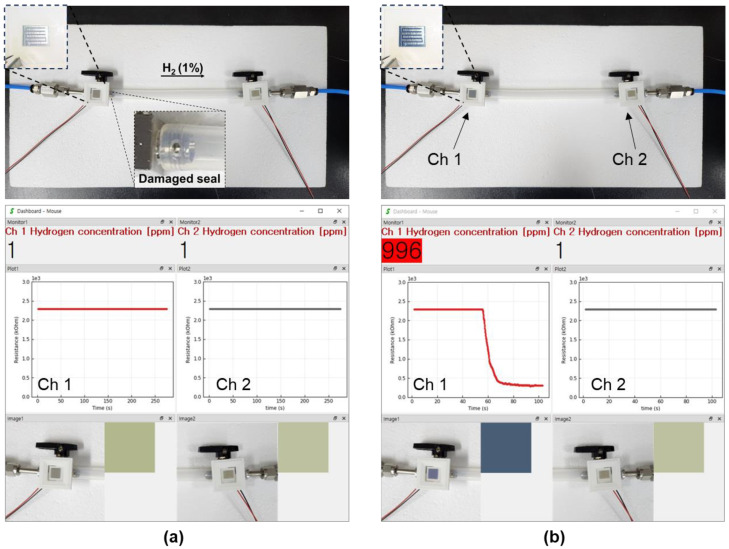
A demonstration to verify the usability of the fabricated hybrid sensor. (**a**) The environment in which hydrogen did not leak and (**b**) the resistance and color change observed after hydrogen leaked.

The sensor we propose is a method that can acquire responsiveness to the same target in two ways using a single sensing material layer. A sensor that exhibits a resistance reaction and a gaschromic reaction to hydrogen at the same time enables anyone or a worker to qualitatively recognize the outflow of hydrogen in a hydrogen handling facility without a signal process. In addition, to manage facilities handling hydrogen, it can be linked with a signal processing system that can quantitatively recognize hydrogen leakage in real time. As an example of this application, a demonstration was conducted to simultaneously observe color change and resistance change in a hydrogen outflow environment. As shown in Figure 13, the sensor was attached to the node where hydrogen leakage from the transmission pipe could occur. Among the two nodes with the ball valve switch in the demonstration, an environment in which hydrogen leaked was intentionally created at the part where the Ch 1 sensor on the left was attached. The sensor connected to Ch 1 of the node where the pipeline was damaged and the sensor connected to Ch 2 of the node where the pipe was not damaged both had similar initial resistance values, and all showed a grayish-white color (Figure 13a). One minute after starting the experiment, hydrogen at a concentration of 1% flowed through the pipe. As shown in Figure 13b, a change in the resistance was observed in Ch 1, where the sensor on the left where the pipeline was damaged was connected; the color of the sensor on the left turned dark blue. Using this simple method, it is possible for a person to observe the outflow of hydrogen with the naked eye or quantitatively analyze the outflow of hydrogen through a signal processing system.

## 4. Conclusions and Future Perspectives

In this study, a hybrid hydrogen sensor that utilizes both chemiresistive and gasochromic reactions was developed as an application case for WO_3_, which has gasochromic characteristics and is among the most widely used MOS materials for gas detection. The fabricated sensor has a structure that can operate as a gasochromic and resistive sensor. In general, MOS materials such as WO_3_ increase carrier and electron mobility as the operating temperature increases and can show higher sensitivity as the depletion layer thickness increases according to the oxide film thickness. However, the gasochromism of WO_3_ can cause a decrease in the amount of color change at high operating temperatures. The dissociated H^+^ ions and electrons migrate to the WO_3_ membrane and undergo redox processes, which leads to the formation of W^5+^ ions. In addition, when a more porous substrate and nano-sized grains are applied, higher sensitivity and gas dissociation, adsorption, and desorption rates can be improved. More improvements were identified in the future applicable methods for forming the gasochromic layer, its structure, and alloy catalysts. The color change expressed by the gasochromic phenomenon can detect hydrogen leakage very intuitively with the human eye, and a sensor that can simultaneously apply the chemiresistive method can detect quantitative hydrogen through a signal processor. The sensor showed a relative resistance change of up to 90% at the same concentration and showed a very high level of color change of ΔE = 18.4. In addition, the resistive response and recovery time of the sensor were 6 and 5 s, respectively, and the gasochromic response and recovery time were approximately 60 and 80 s, respectively. In the demonstration conducted to verify these characteristics, it was possible to successfully verify quantitative and qualitative gas detection using one sensor.

## Figures and Tables

**Table 1:** Result of the EDS spectrum of the Pd/WO_3_ sensing layer.

Element	wt%	wt% Sigma
O	48.04	0.26
Pd	1.43	0.16
W	50.54	0.27
Total:	100.00	

## Data Availability

All data are available from the corresponding author upon request.
